# Supplementary material for: Acetoin Promotes Plant Growth and Alleviates Saline Stress by Activating Metabolic Pathways in Lettuce Seedlings
Source: Plants (Basel). 2024 Nov 26;13(23):3312. doi: 10.3390/plants13233312 (PMC11644064; doi:10.3390/plants13233312)
Supplement: Supplementary file 1 [file plants-13-03312-s001.zip › plants-3200659-supplementary.pdf]

Supplementary material

Supplementary Tables

Table S1. Summary of sequencing data quality

Sample	Raw Reads	Clean Reads	Valid Ratio (%)	Raw Data (bp)	Clean Data (bp)	Q20 (%)	Q30 (%)	GC (%)
CK6-L-1	38804812	38770256	99.91	5820721800	5783190594	96.53	91.04	46.43
CK6-L-2	51374000	51308954	99.87	7706100000	7661231039	97.05	91.86	46.37
CK6-L-3	42922822	42876906	99.89	6438423300	6396600732	96.46	90.94	46.13
AT6-L-1	40768396	40723418	99.89	6115259400	6080707517	97.16	92.11	46.28
AT6-L-2	40421906	40377134	99.89	6063285900	6022354674	96.58	91.11	46.36
AT6-L-3	48221860	48169038	99.89	7233279000	7184033996	96.56	91.04	46.38
CK24-L-1	45624516	45582194	99.91	6843677400	6806486549	96.47	90.94	46.10
CK24-L-2	43355666	43310914	99.90	6503349900	6456725156	96.70	91.34	46.75
CK24-L-3	48971830	48924890	99.90	7345774500	7308313514	96.39	90.67	45.94
AT24-L-1	36151756	36115202	99.90	5422763400	5393307657	96.42	90.85	45.90
AT24-L-2	42446376	42405042	99.90	6366956400	6338063606	96.37	90.74	45.89
AT24-L-3	41022024	40982112	99.90	6153303600	6121931465	96.44	90.88	45.90
CK6-R-1	41682246	41637754	99.89	6252336900	6217895641	96.53	90.96	45.18
CK6-R-2	51274530	51232646	99.92	7691179500	7640815463	97.68	93.12	45.48
CK6-R-3	39824344	39787492	99.91	5973651600	5940079817	96.62	91.26	45.25
AT6-R-1	50646070	50584272	99.88	7596910500	7549812615	97.91	93.72	45.18
AT6-R-2	42282026	42234590	99.89	6342303900	6297251122	96.37	90.76	45.28
AT6-R-3	43829346	43776468	99.88	6574401900	6527540732	96.81	91.56	45.32
CK24-R-1	45740704	45700716	99.91	6861105600	6789778146	96.81	91.50	44.97
CK24-R-2	40875048	40818058	99.86	6131257200	6059490402	96.96	91.69	44.95
CK24-R-3	39787432	39752362	99.91	5968114800	5907517863	96.76	91.38	44.73
AT24-R-1	48444346	48395054	99.90	7266651900	7230952327	96.53	90.92	44.60
AT24-R-2	39262798	39216606	99.88	5889419700	5820362320	97.34	92.51	45.07
AT24-R-3	54619514	54542670	99.86	8192927100	8091121341	96.85	91.44	45.25

Note: CK represents the non-AT treatment control; AT represents the AT treatment; L and R represent the shoots and roots of lettuce seedlings, respectively.

Table S2. Comparison and matching analysis of RNA seq

Sample	Total	Unique Mapped	Ratio (%)	Multiple Mapped (%)	Ratio (%)	Total Mapped	Ratio (%)
CK6-L-1	38747408	34691360	89.53	1704161	4.40	36395521	93.93
CK6-L-2	51285072	47005701	91.66	2257017	4.40	49262718	96.06
CK6-L-3	42867358	38755130	90.41	1742612	4.07	40497742	94.47
AT6-L-1	40710736	37475050	92.05	1700332	4.18	39175382	96.23
AT6-L-2	40369092	36831778	91.24	1602490	3.97	38434268	95.21
AT6-L-3	48140512	43539864	90.44	2097389	4.36	45637253	94.80
CK24-L-1	45570156	41175218	90.36	1827732	4.01	43002950	94.37
CK24-L-2	43295326	39336819	90.86	1886915	4.36	41223734	95.22
CK24-L-3	48909702	44357889	90.69	1983457	4.06	46341346	94.75
AT24-L-1	36103868	32521005	90.08	1419878	3.93	33940883	94.01
AT24-L-2	42389600	38063309	89.79	1716008	4.05	39779317	93.84
AT24-L-3	40964306	36809799	89.86	1672066	4.08	38481865	93.94
CK6-R-1	41633800	37065371	89.03	1371761	3.29	38437132	92.32
CK6-R-2	51229336	46468839	90.71	1747867	3.41	48216706	94.12
CK6-R-3	39783926	35520691	89.28	1403230	3.53	36923921	92.81
AT6-R-1	50580808	46429948	91.79	1836923	3.63	48266871	95.43
AT6-R-2	42229638	37565449	88.96	1489754	3.53	39055203	92.48
AT6-R-3	43772264	39527645	90.30	1524172	3.48	41051817	93.78
CK24-R-1	45696306	41293136	90.36	1639495	3.59	42932631	93.95
CK24-R-2	40813446	36983149	90.62	1454847	3.56	38437996	94.18
CK24-R-3	39748710	35891666	90.30	1358476	3.42	37250142	93.71
AT24-R-1	48391420	43562303	90.02	1644528	3.40	45206831	93.42
AT24-R-2	39212648	35489008	90.50	1367027	3.49	36856035	93.99
AT24-R-3	54538094	49604780	90.95	1919702	3.52	51524482	94.47

Note: CK represents the non-AT treatment control; AT represents the AT treatment; L and R represent the shoots and roots of lettuce seedlings, respectively.

Table S3. KEGG pathway enrichment of differentially expressed genes (DEGs)

	Pathway ID	Pathway	Candidate genes with pathway annotation	Ratio (%)	Pvalue	Qvalue
CK-L VS AT-L	ko01110	Biosynthesis of secondary metabolites	9/18	50%	0.0097	0.0340
(6 h)	ko04075	Plant hormone signal transduction	5/18	28%	0.0021	0.0148
	ko00941	Flavonoid biosynthesis	3/18	17%	0.0004	0.0052
	ko04016	MAPK signaling pathway - plant	3/18	17%	0.0156	0.0404
	ko00350	Tyrosine metabolism	2/18	11%	0.0173	0.0404
	ko00480	Glutathione metabolism	2/18	11%	0.0401	0.0801
	ko00950	Isoquinoline alkaloid biosynthesis	2/18	11%	0.0061	0.0286
CK-R VS AT-R	ko01100	Metabolic pathways	50/81	62%	0.0002	0.0026
(24 h)	ko01110	Biosynthesis of secondary metabolites	42/81	52%	0.0000	0.0000
	ko00940	Phenylpropanoid biosynthesis	15/81	19%	0.0000	0.0000
	ko00071	Fatty acid degradation	7/81	9%	0.0000	0.0005
	ko00350	Tyrosine metabolism	6/81	7%	0.0003	0.0034
	ko01212	Fatty acid metabolism	6/81	7%	0.0064	0.0534
	ko01040	Biosynthesis of unsaturated fatty acids	5/81	6%	0.0005	0.0048
	ko00270	Cysteine and methionine metabolism	5/81	6%	0.0226	0.1459
	ko00909	Sesquiterpenoid and triterpenoid biosynthesis	3/81	4%	0.0110	0.0800
	ko00941	Flavonoid biosynthesis	3/81	4%	0.0271	0.1573
	ko00590	Arachidonic acid metabolism	2/81	2%	0.0444	0.2147
	ko00944	Flavone and flavonol biosynthesis	1/81	1%	0.0345	0.1821
CK-L VS AT-L	ko04626	Plant-pathogen interaction	10/39	26%	0.0001	0.0016
(6 h)	ko04016	MAPK signaling pathway - plant	9/39	23%	0.0000	0.0000
	ko04075	Plant hormone signal transduction	9/39	23%	0.0002	0.0016
	ko00910	Nitrogen metabolism	2/39	5%	0.0272	0.1901
CK-R VS AT-R	ko01110	Biosynthesis of secondary metabolites	10/23	43%	0.0206	0.2898
(24 h)	ko00910	Nitrogen metabolism	2/23	9%	0.0099	0.2898
	ko00071	Fatty acid degradation	2/23	9%	0.0263	0.2898
	ko00561	Glycerolipid metabolism	2/23	9%	0.0481	0.3530

Note: CK represents the non-AT treatment control; AT represents the AT treatment; L and R represent the shoots and roots of lettuce seedlings, respectively.

Table S4. The level of gene expression (up/down) related to plant hormone under the acetoin (AT) treatment in lettuce

Plant hormone	Gene name	CK6-L-vs-AT6-L	CK24-L-vs-AT24-L	Related gene in lettuce
Ethylene	*CTR1*	0	1	ncbi_11190657
	EBF1/EBF2	2	1	ncbi_111917430,<br>ncbi_111921717
ABA	*PYL4*	1	0	ncbi_111879857
	PP2CA	0	1	ncbi_111910618

Note: CK6/24 represents the non-AT control at 6 h (24 h post treatment) and AT6/24 represents the AT control at 6 h (24 h post treatment); L and R represent the shoots and roots of lettuce seedlings, respectively.

Table S5. Cascade pathway of MAPK and plant hormone signal as well as significant differentially expressed genes

Cascade pathway in our study	CK6-L-vs-AT6-L	CK24-L-vs-AT24-L	Related genes in lettuce
Ethylene→CTR1→MKK9→MPK3/MPK6	2	2	ncbi_111917430, ncbi_111921717, ncbi_111906571, ncbi_111915142
ABA→PYL/PYR→PP2C→SnRK2→MAPKKK17/18→MKK3→MPK1/2/7/14	1	1	ncbi_111879857, ncbi_111910618
Ca^{2+}→CAM→MPK8/MAPKKK→MKK3→MPK8	0	2	ncbi_111914172, ncbi_111920042

Note: CK6/24 represents the non-AT control at 6 h (24 h post treatment) and AT6/24 represents the AT control at 6 h (24 h post treatment); L and R represent the shoots and roots of lettuce seedlings, respectively.

Table S6. Relative expression level of 10 genes with response to acetoin (AT) application measured using qRT-PCR ($2^{-\Delta\Delta Ct}$)

Gene name	6 h				24 h			
	CK-L	AT-L	CK-R	AT-R	CK-L	AT-L	CK-R	AT-R
LsPYL4	1.10±0.18[b]	3.00±0.14[a]	1.01±0.06[b]	1.39±0.05[a]	1.02±0.10[a]	1.02±0.08[a]	1.03±0.11[a]	0.72±0.05[a]
LsEBF2	1.11±0.23[a]	1.32±0.06[a]	1.01±0.06[a]	1.36±0.06[a]	1.05±0.16[a]	0.33±0.02[b]	1.02±0.10[a]	0.92±0.12[a]
LsMKK9	1.09±0.17[b]	3.01±0.21[a]	1.02±0.09[a]	1.33±0.05[a]	1.08±0.20[a]	0.47±0.04[b]	1.02±0.10[a]	0.67±0.06[a]
LsCP1	1.09±0.19[b]	1.95±0.12[a]	1.01±0.05[a]	1.11±0.05[a]	1.13±0.25[a]	0.58±0.03[b]	1.03±0.12[a]	0.50±0.07[b]
LsCTR1	1.11±0.23[a]	1.33±0.09[a]	1.01±0.05[a]	1.22±0.03[a]	1.01±0.06[a]	0.52±0.02[b]	1.00±0.04[a]	0.51±0.06[b]
LsCML35	1.07±0.16[b]	1.49±0.09[a]	1.00±0.04[a]	1.19±0.06[a]	1.09±0.21[a]	0.69±0.05[b]	1.02±0.09[a]	0.50±0.08[b]
LsCML39	1.19±0.31[b]	1.99±0.21[a]	1.06±0.15[a]	1.54±0.08[a]	1.32±0.45[a]	0.53±0.08[b]	1.10±0.22[a]	1.26±0.22[a]
LsCML41	1.09±0.20[a]	1.17±0.06[a]	1.00±0.04[a]	1.26±0.08[a]	1.06±0.14[a]	0.68±0.10[b]	1.02±0.08[a]	0.55±0.06[b]
LsCML46	1.11±0.20[b]	3.45±0.17[a]	1.08±0.16[a]	1.14±0.14[a]	1.10±0.23[a]	0.64±0.06[a]	1.05±0.14[a]	0.60±0.14[a]
LsPP2CA	1.07±0.15[b]	1.88±0.16[a]	1.00±0.04[b]	1.34±0.06[a]	1.03±0.12[a]	0.85±0.07[a]	1.03±0.12[a]	0.50±0.04[b]
LsGapC	1.00±0.05[a]	0.99±0.08[a]	1.01±0.06[b]	1.21±0.04[a]	1.00±0.04[a]	1.07±0.04[a]	1.00±0.01[a]	0.94±0.04[a]
LsActin	1.00±0.03[a]	1.12±0.05[a]	1.00±0.03[a]	0.93±0.04[a]	1.00±0.03[a]	0.97±0.02[a]	1.00±0.01[a]	1.02±0.04[a]
LsTublin	1.01±0.06[a]	0.94±0.06[a]	1.00±0.04[a]	0.90±0.04[a]	1.00±0.03[a]	0.97±0.02[a]	1.00±0.02[a]	1.05±0.03[a]

Note: CK represents the non-AT treatment control; AT represents the AT treatment; L and R represent the shoots and roots of lettuce seedlings, respectively.

Supplementary Figures
Figure S1

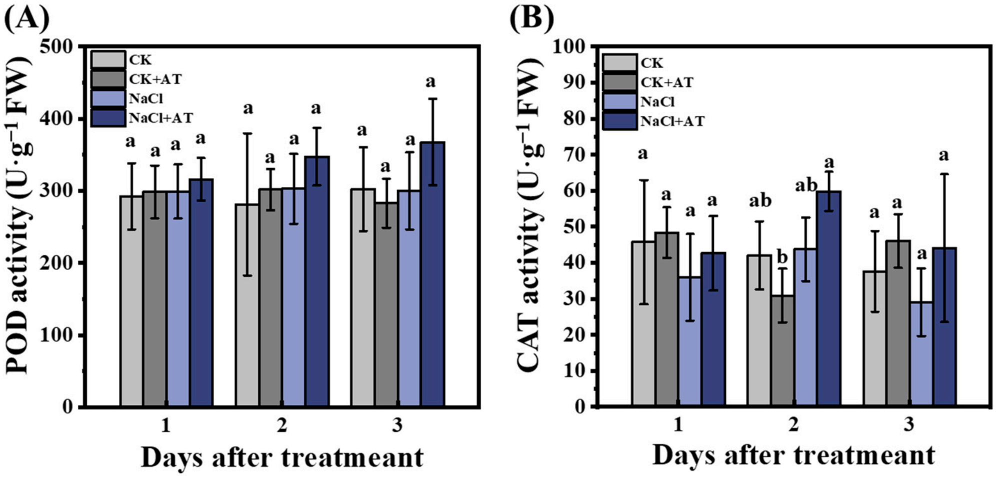

Figure S1. Dynamic changes of the defense enzyme activities in leaves of lettuce seedlings under non-saline and saline conditions. **(a)** Peroxidase (POD) activity, and **(b)** catalase (CAT) activity under CK (non-AT and non-NaCl treatment control), CK + AT, NaCl (3 g NaCl per kg of soil), and NaCl + AT treatments. Different letters indicate significant difference ($P < 0.05$) according to an independent-sample *T* test.

Figure S2

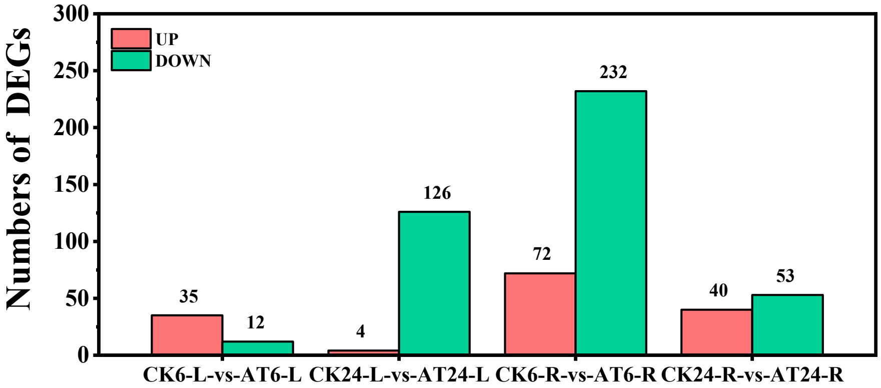

Figure S2. Numbers of upregulated and downregulated genes induced by acetoin. CK6/24 represents the non-AT control at 6 h (24 h post treatment) and AT6/24 represents the AT control at 6 h (24 h post treatment); L and R represent the shoots and roots of lettuce seedlings, respectively; and DEGs represents differentially expressed genes.
